# Annealing effect and photovoltaic properties of nano-ZnS/textured *p*-Si heterojunction

**DOI:** 10.1186/1556-276X-8-470

**Published:** 2013-11-09

**Authors:** Liang-Wen Ji, Yu-Jen Hsiao, I-Tseng Tang, Teen-Hang Meen, Chien-Hung Liu, Jenn-Kai Tsai, Tien-Chuan Wu, Yue-Sian Wu

**Affiliations:** 1 Institute of Electro-Optical and Materials Science, National Formosa University, Yunlin 632, Taiwan; 2 National Nano Device Laboratories, Tainan 741, Taiwan; 3 Department of Greenergy Technology, National University of Tainan, Tainan 700, Taiwan; 4 Department of Electronic Engineering, National Formosa University, Yunlin 632, Taiwan; 5 Department of Mechanical Engineering, National Chung-Hsing University, Taichung 402, Taiwan

**Keywords:** Heterojunction, Nanocrystal, ZnS

## Abstract

The preparation and characterization of heterojunction solar cell with ZnS nanocrystals synthesized by chemical bath deposition method were studied in this work. The ZnS nanocrystals were characterized by X-ray diffraction (XRD) and high-resolution transmission electron microscopy (HRTEM). Lower reflectance spectra were found as the annealing temperature of ZnS film increased on the textured *p*-Si substrate. It was found that the power conversion efficiency (PCE) of the AZO/ZnS/textured *p*-Si heterojunction solar cell with an annealing temperature of 250°C was *η* = 3.66%.

## Background

Recently, 2D nanostructure P-N junctions have attracted a great deal of attention for their potential applications in photovoltaic device [1]. Zinc sulfide (ZnS) was one of the first semiconductors discovered [2] and is also an important semiconductor material with direct wide band gaps for cubic and hexagonal phases of 3.72 and 3.77 eV, respectively [3]. It has a high absorption coefficient in the visible range of the optical spectrum and reasonably good electrical properties [4]. This property makes ZnS very attractive as an absorber in heterojunction thin-film solar cells [5,6]. Furthermore, ZnS also offers the advantage of being a nontoxic semiconductor material (without Cd and Pb). A cell with ITO/PEDOT:PSS/P3HT:ZnS/Al structure was obtained by Bredol et al. [7], which showed a very high open-circuit voltage (*V*_oc_) value of 1.2 V and a power conversion efficiency of 0.2%.

In recent years, ZnS thin films have been grown by a variety of deposition techniques, such as chemical bath deposition [8], evaporation [9], and solvothermal method [10]. Chemical bath deposition is promising because of its low cost, arbitrary substrate shapes, simplicity, and capability of large area preparation. There are many reports of successful fabrication of ZnS-based heterojunction solar cells by the chemical bath deposition method, such as with CIGS used for the n-type emitter layer [11].

This study aimed to grow ZnS thin films on a p-type silicon wafer using chemical bath deposition method. Crystalline silicon solar cells are presently due to their higher photovoltaic conversion efficiency, long-term stability, and optimized manufacturing process [12]. *n*-ZnS/textured *p*-Si heterojunctions were produced, and their photovoltaic properties were investigated under various annealing temperatures.

## Methods

ZnS nanocrystals were prepared using the chemical bath deposition (CBD) procedure. Aqueous solutions of 0.15 M ZnSO_4_, 0.5 M thiourea (NH_2_)_2_CS, and 0.2 M ammonia NH_3_ were mixed in a glass beaker under magnetic stirring. The beaker was maintained at a reaction temperature of 80°C using a water bath for 30 min. In addition, the silicon wafer samples were cleaned using a standard wet cleaning process. Subsequently, KOH was diluted to isotropically etch the silicon wafer to form a surface with a pyramid texture [13].

The preparation process of ZnS/textured *p*-Si solar cells has three parts: Firstly, square samples of 1.5 × 1.5 cm^2^ were cut from a (100)-oriented p-type silicon wafer with *ρ* = 1–10 Ω cm and thickness of 200 μm. For ohmic contact electrodes, DC sputtering was used to deposit about 2 μm of Al onto the back of the Si substrates, followed by furnace annealing at 450°C for 1 h in Ar ambient to serve as the *p*-ohmic contact electrodes. Secondly, a 200-nm n-type ZnS thin film was deposited on the prepared p-type Si by chemical bath deposition in order to form a ZnS/*p*-Si heterojunction. Finally, an AZO film and Al metal grid with a thickness of about 0.4 and 2 μm, respectively, were deposited by sputtering.

The phase identification was performed by X-ray powder diffraction (Rigaku Dmax-33, Rigaku Corporation, Tokyo, Japan). The morphology and microstructure were examined by high-resolution transmission electron microscopy (HRTEM) (HF-2000, Hitachi, Tokyo, Japan). The reflectance spectra were measured at room temperature using a JASCO UV-670 UV–vis spectrophotometer (Jasco Analytical Instruments, Easton, MD, USA). The current–voltage measurements (Keithley 2410 source meter, Keithley Instruments Inc., Cleveland, OH, USA) were obtained using a solar simulator (Teltec, Mainhardt, Germany) with an AM 1.5 filter under an irradiation intensity of 100 mW/cm^2^.

## Results and discussion

**Figure 1:**
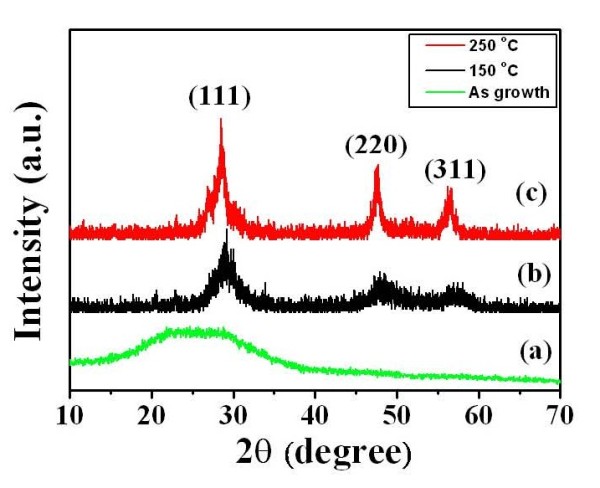
**XRD spectra of the ZnS films.** Grown (spectrum a) without annealing and at annealing temperatures of (spectrum b) 150°C and (c) 250°C, respectively.

X-ray diffraction (XRD) patterns of ZnS grown without annealing and at annealing temperatures of 150°C and 250°C are shown in Figure 1. ZnS formed directly from the amorphous precursor using chemical bath deposition. All of the peaks for various annealing temperatures were identified to be those of the cubic ZnS phase (JCPDS card no. 79–0043) [14]. The crystallinity of ZnS increased along with annealing temperature. When the temperature was increased to 250°C, the peaks of (111), (220), and (311) were obviously seen. In this experiment, as ZnSO_4_ was dissolved in water, Zn^2+^ ions could form a variety of complexes in the solution, and this was hydrolyzed to form Zn(OH)_2_. The possible chemical reactions for the synthesis of ZnS nanocrystals are as follows:

(1) $${\left[\mathrm{Zn}{\left(H_{2}O\right)}_{5}\left(\mathrm{OH}\right)\right]}^{+}+H^{+}↔\mathrm{Zn}{\left(\mathrm{OH}\right)}_{2}+2H^{+}$$

(2) $${\mathrm{CH}}_{3}{\mathrm{CSNH}}_{2}+H^{+}+2H_{2}O↔H_{2}S+{\mathrm{CH}}_{3}\mathrm{COOH}+{{\mathrm{NH}}_{4}}^{+}$$

(3) $$H_{2}S↔{\mathrm{HS}}^{-}+H^{+},{\mathrm{HS}}^{-}↔S^{2-}+H^{+}$$

(4) $${{\mathrm{Zn}}^{2}}^{+}+S^{2-}\to \mathrm{ZnS}$$

During the reaction processes, sulfide ions release slowly from CH_3_CSNH_2_ and react with zinc ions. Consequently, ZnS nanocrystals form via an *in situ* chemical reaction manner. Equation 4 indicates that ZnS is produced by the reaction of S^2-^ and Zn^2+^.

**Figure 2:**
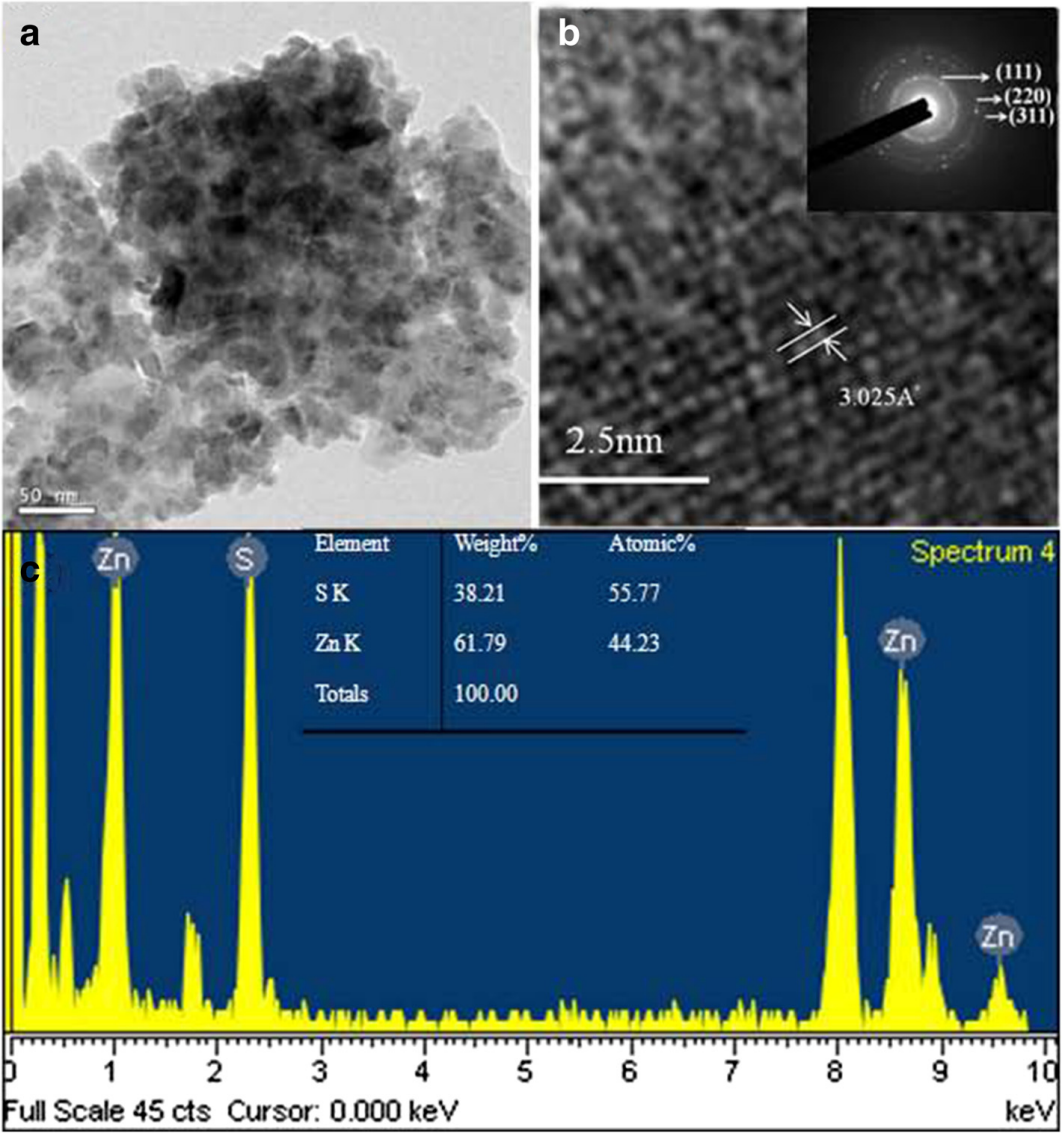
**Structural properties of as-synthesized ZnS nanocrystals. (a)** TEM image of as-synthesized ZnS nanocrystals. **(b)** HRTEM image of the nanocrystal and the electron diffraction pattern. **(c)** EDS analysis of the ZnS nanocrystals.

TEM analysis provides further insights into the structural properties of as-synthesized ZnS nanocrystals. Figure 2a shows a low-magnification TEM image where the nanocrystals are clearly observed. The average grain size of the ZnS nanocrystal was about 16 nm. The crystalline ZnS were identified by the electron diffraction (ED) pattern in the inset of Figure 2b, which shows diffused rings indicating that the ZnS hollow spheres are constructed of polycrystalline ZnS nanocrystals. The concentric rings can be assigned to diffractions from the (111), (220), and (311) planes of cubic ZnS, which coincides with the XRD pattern. A representative HRTEM image enlarging the round part of the structure in Figure 2b is given. The interplanar distances of the crystal fringes are about 3.03 Å. The energy-dispersive X-ray spectroscopy (EDS) line profiles indicate that the nanocrystal consists of Zn and S, as shown in Figure 2c. In addition, the atomic concentrations of Zn = 56% and S = 44% were calculated from the EDS spectrum.

**Figure 3:**
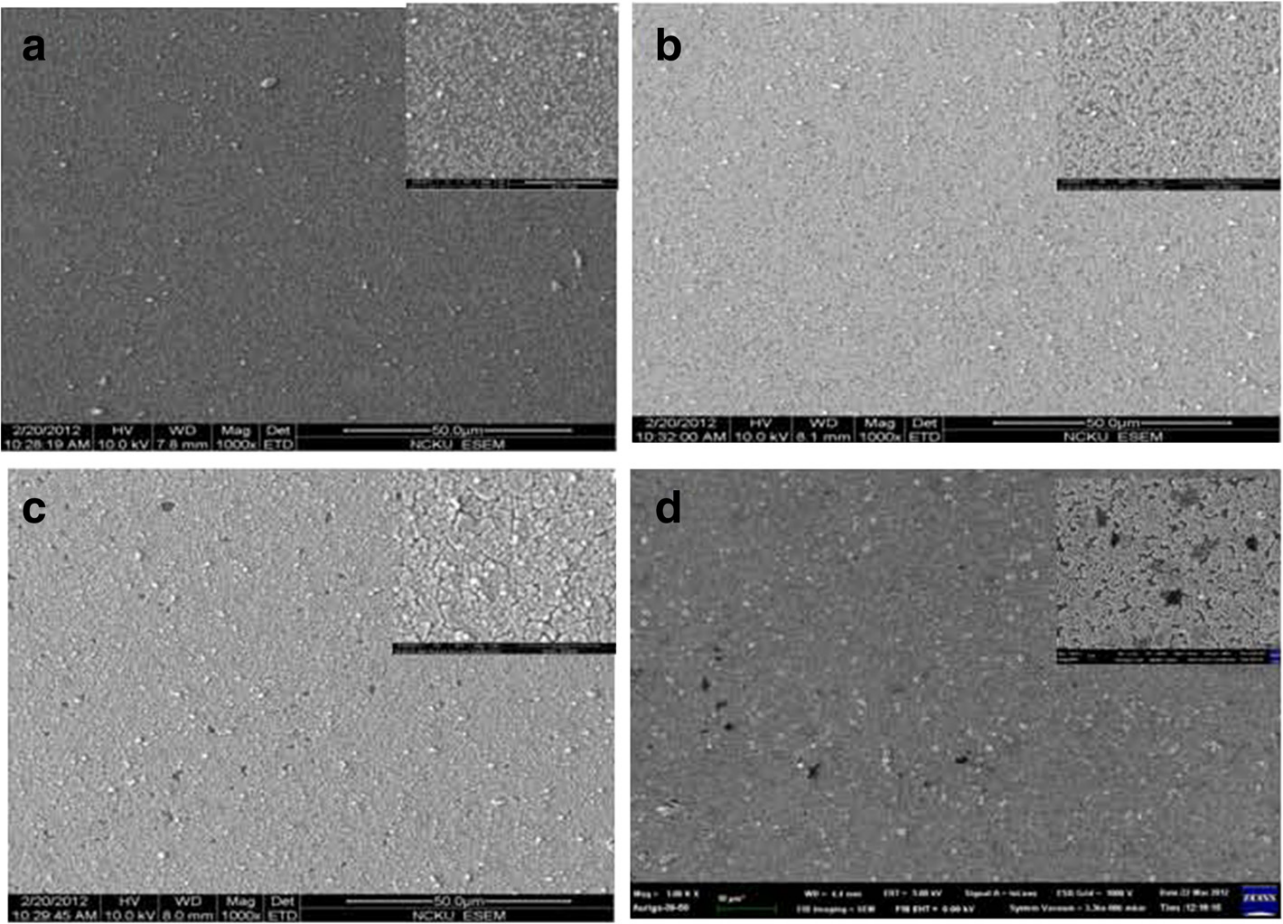
**SEM images of the ZnS film annealed at different temperatures. (a)** 100°C, **(b)** 150°C, **(c)** 200°C, and **(d)** 250°C, respectively.

Figure 3a,b,c,d shows scanning electron microscopy (SEM) images of the ZnS film on Si plane annealed at temperatures of 100°C, 150°C, 200°C, and 250°C, respectively. It can be clearly seen that the dominant feature of the films is the appearance of small islands. The grain particles were condensed by assembled nanocrystals. It was conjectured that the assembly effect arising from nanocrystals are responsible for the decrease of surface energy. The particle size increased as the sintering temperature increased. It is believed that a higher temperature enhanced higher atomic mobility and caused faster grain growth.

**Figure 4:**
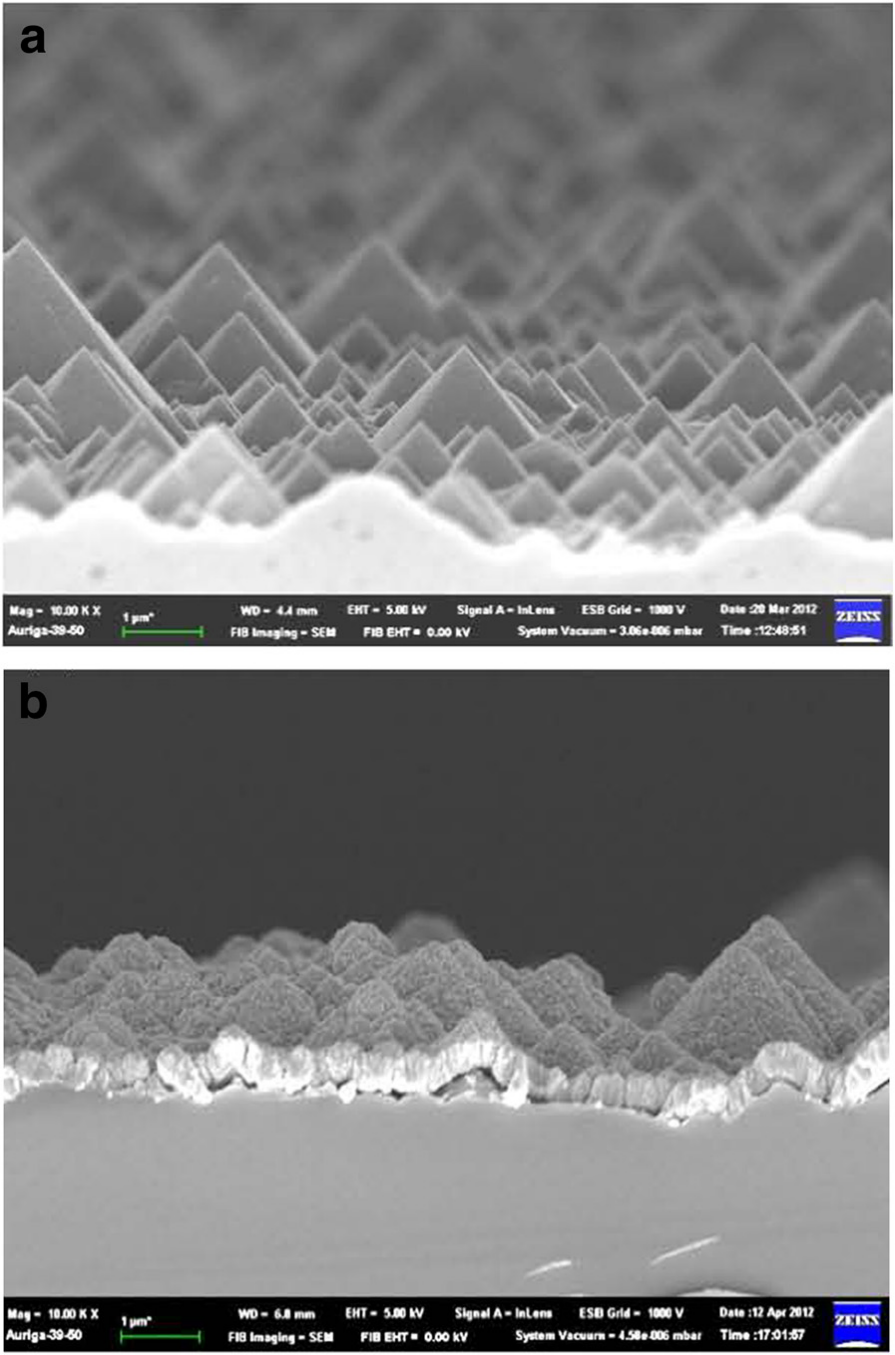
**SEM images of the textured *p*-Si substrate. (a)** Side-view SEM images of the textured *p*-Si substrate and **(b)** cross section of the AZO/ZnS/textured *p*-Si layer.

Figure 4a shows side-view SEM images of the textured *p*-Si substrate produced using wet etching. Uniform pyramids were grown on the surface of the *p*-Si, and these function as antireflective structures. ZnS films were grown on the surface of the textured *p*-Si substrate with thicknesses of about 200 nm. The cross-sectional images of the ZnS/textured *p*-Si substrate exhibit a rough surface in Figure 4b.

**Figure 5:**
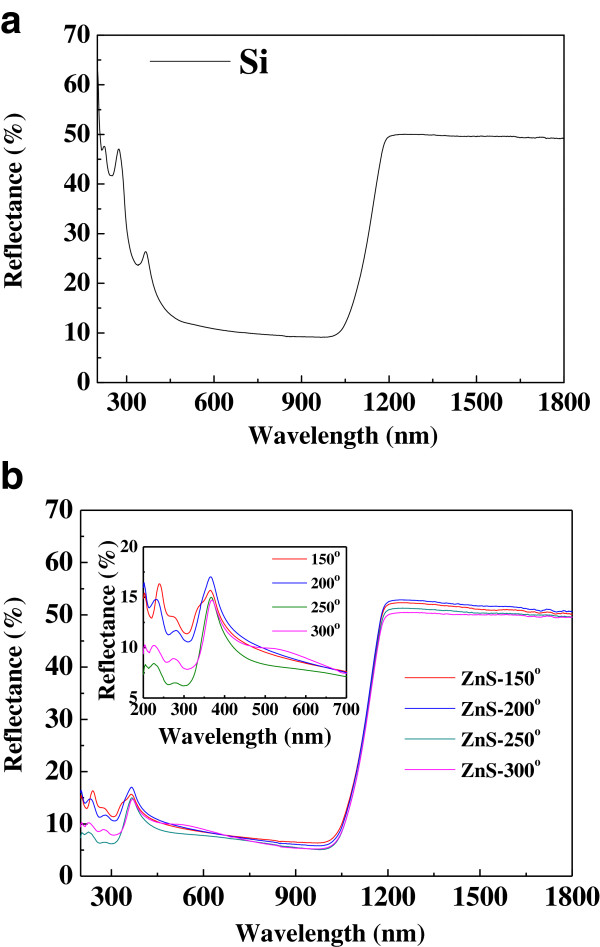
**Reflectance spectra. (a)** The textured *p*-Si and **(b)** the ZnS film annealed at various temperatures on textured *p*-Si substrate.

Figure 5a,b shows the reflectance spectra of the textured *p*-Si and the ZnS film annealed at various temperatures on textured *p*-Si substrate in the range of 300 to 1,000 nm. The average reflectance was about 8.8%, 8.7%, 7.6%, and 8.1% for the ZnS films on the textured *p*-Si substrate with annealing temperatures of 150°C, 200°C, 250°C, and 300°C, respectively. These values are lower than those for the textured *p*-Si, with an average reflectance of about 12.7%. Therefore, the reflectance can significantly be reduced by depositing the ZnS film on textured Si substrate. This can be attributed to the decreasing reflectance of the ZnS film at short wavelengths or the surface coating decreasing the reflectance [15].

**Figure 6:**
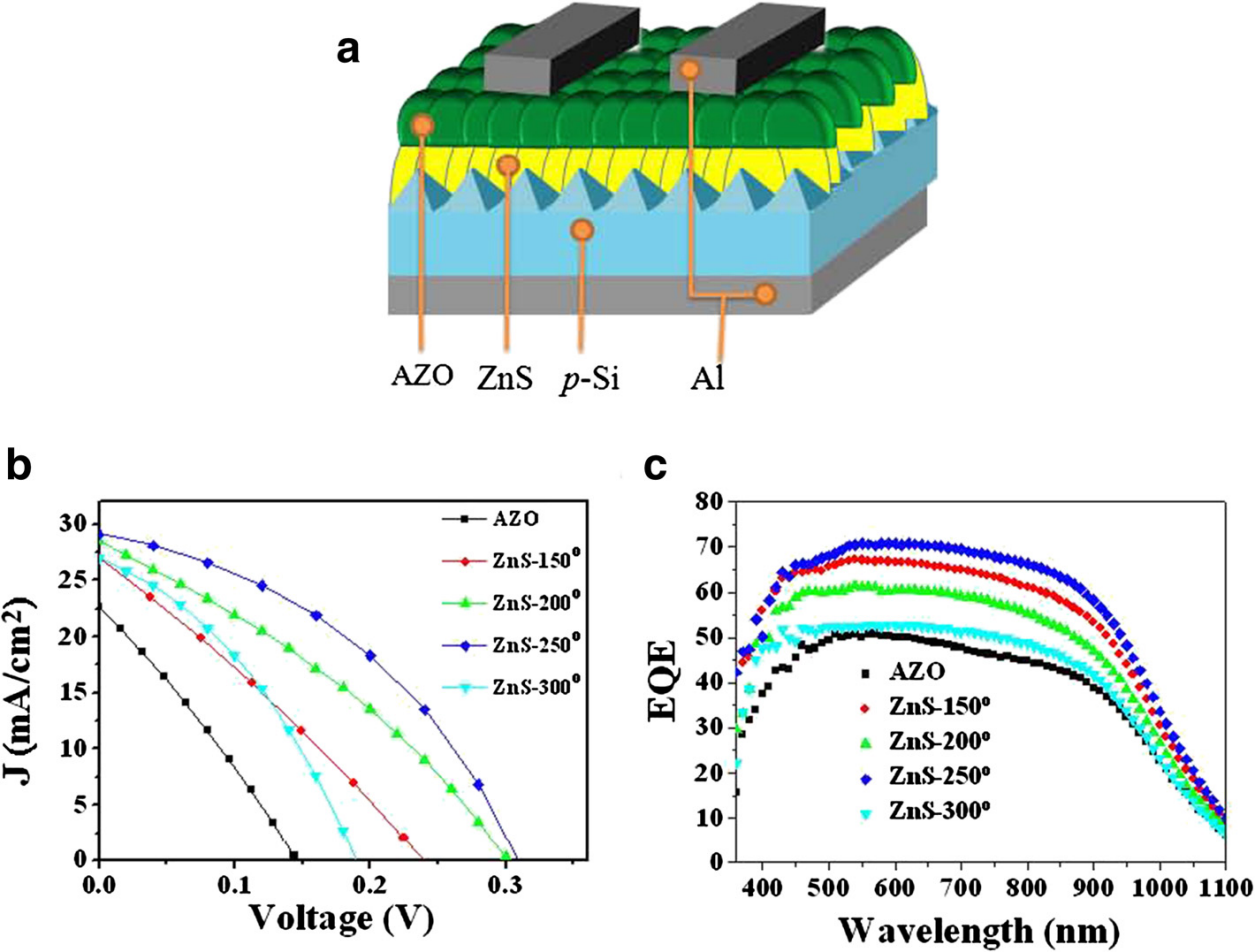
**Structure and characteristics of the heterojunction device. (a)** Schematic diagram of the ZnS/textured *p*-Si heterojunction solar cell. **(b)***J*-*V* characteristics and **(c)** the EQE spectra of the ZnS/textured *p*-Si heterojunction solar cell with various annealing temperatures.

Figure 6a shows the structure of the heterojunction device in which the ZnS/textured *p*-Si was the photoactive layer. The photovoltaic characteristics of the AZO/ZnS/textured *p*-Si heterojunction device with ZnS film annealed at various temperatures are given in Table 1. The characteristic of the AZO/ZnS film deposited on textured *p*-Si substrate was studied for the first time in this work. The deposition thickness of AZO was close to 400 nm and exhibits good coverage on the *p*-Si substrate. Jiang et al. [16] fabricated SnS/α-Si heterojunction photovoltaic devices, and the junction exhibited a typical rectifying diode behavior with a short-circuit current density of 1.55 mA/cm^2^. Therefore, the AZO/ZnS/textured *p*-Si structure is suitable for use in solar cells in this study.

**Table 1:** **Photovoltaic performance of the AZO/ZnS/textured *p*-Si heterojunction solar cell with various annealing temperatures**

**Device**	***V***_**oc**_	***J***_**sc **_**(mA/cm**^**2**^**)**	**FF (%)**	**Efficiency (%)**
No ZnS	0.139	22.53	28.50	0.89
ZnS (150°C)	0.239	26.97	29.38	1.90
ZnS (200°C)	0.299	28.55	32.60	2.79
ZnS (250°C)	0.319	29.11	39.31	3.66
ZnS (300°C)	0.179	26.55	23.42	1.94

Under AM 1.5 G at 100 mW/cm^2^ illumination. FF, fill factor.

The current density-voltage (*J*-*V*) characteristics of the finished photovoltaic devices were measured under an illumination intensity of 100 mW/cm^2^ and shown in Figure 6b. The measurements show that the ZnS film deposited onto the *p*-Si results in increased *V*_oc_. The power conversion efficiency (PCE) of the devices improved significantly from 0.89% to 3.66% when the ZnS film annealing temperature was 250°C. The highest *V*_oc_ was 0.32 V and the highest current density was 29.1 mA/cm^2^. Therefore, the best annealing temperature of the ZnS film is 250°C, with a PCE of 3.66%. When the annealing temperature of the ZnS film increased to 300°C, the efficiency decreased because of a large percentage decrease in *V*_oc_. The possible reason is that the ZnS film included impurities or defects originating from high-temperature process. In addition, the value of *R*_sh_ has relatively changed, resulting in element composition instability. Therefore, *V*_oc_ and cell performance deteriorated with a 300°C annealing process. A similar phenomenon was also observed in the ILGAR-ZnO layers to cover the rough CIGSSe absorber heterojunction thin-film solar cells [17]. Therefore, the interface of the AZO/ZnS/textured *p*-Si heterojunction may have some defects at higher annealing temperature of ZnS films, and this decreases the PCE. The external quantum efficiency (EQE) spectra for the photovoltaic devices of the AZO/ZnS/ textured *p*-Si heterojunction solar cell are shown in Figure 6c. All EQE spectra are similar in shape, except for the sample without ZnS, and the EQE value for the optimal annealing temperature of the ZnS film (250°C) is higher than that of most wavelengths. The differences in the EQE spectra are due to the increase in leakage current that occurs by decreasing the FF, and therefore, the interface of the AZO/ZnS/textured *p*-Si heterojunction may have some defects for ZnS films annealed at higher temperature.

## Conclusions

A chemical bath deposition method for the synthesis of ZnS nanocrystals is reported in this work. The cubic ZnS film was deposited on *p*-Si substrate and obtained a well-crystallized single phase with various annealing temperatures. Lower reflectance spectra were found as the annealing temperature of ZnS film increased on the textured *p*-Si substrate. The photovoltaic characteristics of the AZO/ZnS/textured *p*-Si heterojunction solar cells with various annealing temperatures of the ZnS film were examined, and the In_2_S_3_ film with an annealing temperature at 250°C had *η* = 3.66% under an illumination of 100 mW/cm^2^.

## Competing interests

The authors declare that they have no competing interests.

## Authors’ contributions

LWJ and YJH carried out the design of the study and drafted this manuscript. ITT, THM, and CHL conceived of the study and participated in its design and coordination. JKT, TCW, and YSW carried out the preparation of the samples and characteristic measurements. All authors read and approved the final manuscript.
